# Trauma risk score matching for observational studies in orthopedic trauma dataset and code

**DOI:** 10.1016/j.dib.2022.107794

**Published:** 2022-01-05

**Authors:** Rown Parola, Abhishek Ganta, Kenneth A. Egol, Sanjit R. Konda

**Affiliations:** a NYU Langone Orthopedic Hospital, 301 E 17th St, New York NY 10010, United States; b Jamaica Hospital Medical Center, 8900 Van Wyck Expy, Richmond Hill, NY 11418, United States

**Keywords:** Propensity Score Matching, Propensity Matching, Orthopedic Trauma, Research Methods, Trauma Risk Score, Database Analytics, Hip fracture, Geriatric Trauma

## Abstract

The dataset presented was collected via retrospective review from an orthopedic trauma database approved by the institutional review board at the author's institution from patients treated at any of the four hospitals serviced by the academic orthopedic surgery department. Femoral neck and intertrochanteric hip fracture patients from low energy mechanisms admitted between October 2014 and February 2020, were selected if they were age 55 or older and had recorded sex, body mass index (BMI), Charlson Comorbidity Index (CCI), American Society of Anaesthesiologists (ASA) physical status classification, Glasgow Coma Score, Abbreviated Injury Severity score for the chest, head and neck, and extremities, and ambulation status prior to injury.

The resultant 1,590 subject dataset may be analysed via the supplied R statistical code to determine the frequency of equipoise in baseline and outcome variables from propensity matching via three matching schemes. The code implements three matching schemes including matching by (1) The Score for Trauma Triage in Geriatric and Middle-Aged (STTGMA) (2) CCI alone, or (3) a combination of sex, age, CCI and BMI. The code selects a subset of ten percent of hip fracture patients by a pseudorandom number generator (PRNG). The code matches the remaining patients 1:1 to the selected patients by propensity score generated by logistic regression of STTGMA, CCI, or a combination of sex, age, CCI and BMI using greedy nearest neighbor matching without replacement by the MatchIt package for R software. The code then compares matched cohorts by Chi-square, Fisher, or Mann-Whitney U test with significance level of 0.05 representing a 5% chance of significant differences due to random sampling of subjects.

The supplied code repeats the random selection, matching and testing process 100,000 times for each matching method. The resultant code output is the frequency of significantly different demographic or outcome parameters among matched cohorts by matching method.

This data and statistical code have reuse potential to explore alternative matching schemes. The supplied baseline variables should be robust enough to derive alternative risk scores for each patient which may be included as a matching variable for comparison. The authors also look forward to unexpected ways that this data may be used by readers.

## Specifications Table

**Table:** 

Subject	Orthopaedics, Sports Medicine and Rehabilitation
Specific subject area	Orthopaedic Trauma
Type of data	Database R Statistical Code
How the data were acquired	The data presented was collected via retrospective review from an orthopedic trauma database approved by the institutional review board at the author's institution from patients treated at any of the four hospitals serviced by the academic orthopedic surgery department. Data was abstracted from the electronic medical records of each subject.
Data format	Raw – The R statistical code is presented for use in analysing the supplied database and reproducing the tables in the original research article. Raw – The orthopedic trauma hip fracture database described above prior to filtering for subjects matching inclusion criteria (done within the supplied R code) and shared as an excel spreadsheet. Both items may be found at the following repository: Parola, Rown (2021), “STTGMA Matching Dataset”, Mendeley Data, V1, doi:10.17632/d5b5rx6vmf.1 and published at: https://data.mendeley.com/datasets/d5b5rx6vmf/1
Description of data collection	Femoral neck and intertrochanteric hip fracture patients from low energy mechanisms admitted between October 2014 and February 2020, were selected if they were age 55 or older and had recorded sex, body mass index, Charlson Comorbidity Index, American Society of Anaesthesiologists physical status classification, Glasgow Coma Score, Abbreviated Injury Severity score for the chest, head and neck, and extremities, and ambulation status prior to injury.
Data source location	• *Institution: NYU Langone Orthopedic Hospital* • *City/Town/Region: New York, NY* • *Country: USA*
Data accessibility	Repository name: Mendeley Data Direct URL to data: https://data.mendeley.com/datasets/d5b5rx6vmf/1
Related Research Article	R. Parola, A. Ganta, K.A. Egol, S.R. Konda, Trauma Risk Score Matching for Observational Studies in Orthopedic Trauma, Injury. (2021). https://doi.org/10.1016/j.injury.2021.12.009.

## Value of the Data

• These data are useful for testing propensity matching schemes in a typical hip fracture population.
• This data may benefit orthopedic trauma researchers wishing to employ or improve propensity matching schemes
• This data may be used by deriving different propensity matching schemes from the baseline variables to test against the provided three methods.
• This data may be used to investigate correlations within a typical hip fracture population.

## 1 Data Description

STTGMA Matching DB.xlsx is an excel file that contains the raw data database of hip fracture subjects. Several baseline and outcome variables are collected, with entries further described in the Data Dictionary tab. The data is structured with each row representing a single subject and each column containing a baseline, surgical, or outcome variable corresponding to the subject row.

STTGMA Matching.R is an R statistical programming script for use with the R language. In addition to having R installed, the script also uses the R packages readxl, tidyverse, openxlsx, arsenal, and MatchIt. This commented file analyses the data in the STTGMA Matching DB.xlsx file. Matching variables used in the experiment include Charlson Comorbidity Index (CCI) [1] in column Q, sex in column H, Age in column G, body mass index (BMI) in column AB, and the appropriate Score for Trauma Triage in Geriatric and Middle-Aged (STTGMA) score [2], [3], [4] in column BM based on injury mechanism energy in column L.

## 2 Experimental Design, Materials and Methods

An Institutional Review Board approved hip fracture database was queried for any patient aged 55 and older treated surgically after sustaining a femoral neck or intertrochanteric [AO/OTA 31A or 31B] hip fracture. Between October 2014 and February 2020, all patients treated at 4 hospitals within a single academic medical center were analyzed. All patients were treated by staff and resident surgeons.

Information regarding baseline demographics and injury status at presentation were retrospectively reviewed through electronic medical records. All patients who met inclusion criteria were included in the final study analysis. Demographic and clinical variables collected included patient sex, age, body mass index (BMI), pre-injury ambulatory status, comorbidities as measured by the CCI, and physiologic status as measured by the ASA physical status classification system. Fractures were classified according to the system of the Orthopedic Trauma Association (AO/OTA) [5]. Recorded injury details included Glasgow Coma Scale (GCS) at presentation and Abbreviated Injury Severity score for the head and neck (AIS-HN), chest (AIS-C), and pelvis and extremity (AIS-EXT).

Minor complication reviewed included postoperative acute kidney injury (AKI). Major complications reviewed included sepsis or septic shock, pneumonia, acute respiratory failure, stroke, myocardial infarction (MI), cardiac arrest, deep vein thrombus, and pulmonary embolism. Inpatient, 30-day and 1-year mortality were also reviewed. Hospital quality measure reviewed included need for ICU admission and readmission at 30- and 90-days post discharge.

Ten percent of hip fracture patients were selected by a pseudorandom number generator (PRNG) [6]. The remaining patients were matched 1:1 to the selected patients by propensity score generated by logistic regression of STTGMA, CCI, or a combination of sex, age, CCI and BMI using greedy nearest neighbor matching without replacement by the MatchIt package for R software version 4.02 [7]. Matched cohorts were compared by Chi-square, Fisher, or Mann-Whitney U test with significance level of 0.05 representing a 5% chance of significant differences due to random sampling of subjects.

The supplied code repeats the random selection, matching and testing process 100,000 times for each matching method. Weighting of propensity score components in the combination matching method are optimized for each matched cohort. Cumulative totals of significantly different matched cohort comparisons were summed for STTGMA, CCI and combination matching methods. The resultant output is the frequency of significantly different demographic or outcome parameters among matched cohorts by matching method.

## Ethics Statements

This work was approved by our institutional review board (IRB) which is in accordance with The Code of Ethics of the World Medical Association (Declaration of Helsinki), the corresponding IRB number is s20-001766. The IRB granted this study a waiver of authorization/informed consent. The waiver was granted due to the deemed minimal risk involved in retrospectively collecting deidentified patient data to populate the dataset.

## CRediT authorship contribution statement

**Rown Parola:** Conceptualization, Methodology, Software, Data curation, Writing – original draft, Writing – review & editing. **Abhishek Ganta:** Writing – review & editing. **Kenneth A. Egol:** Writing – review & editing. **Sanjit R. Konda:** Conceptualization, Writing – review & editing.

## Declaration of Competing Interest

The authors declare that they have no known competing financial interests or personal relationships that could have appeared to influence the work reported in this paper.

## Appendix Supplementary materials

Image, application 1

Image, application 2
